# Letter from the U. S. Navy

**Published:** 1863-08

**Authors:** J. F. Norton

**Affiliations:** Surgeon U. S. Brig Perry


### ART. II.—*Letter from the U. S. Navy*—By J. F. Norton.

U. S. Brig Perry, June 17, 1863.

Dr. Miner:

Sir:—In communicating the following, not so much for its intrinsic value, as to assure yourself and readers of the Journal, that I have not at least become insensible to the duty and obligation naturally devolving upon me, as a member of our time-honored profession. With our complement of men, who number all told, about 100, we have had some 500 sick days for the last four months. This would appear unsusually large but from the fact that a portion of this number were old constitutional cases, such as strumous, indolent and syphilitic ulcers. These have all occurred upon those of a scrofulous habit, who, upon receiving a slight wound upon the limb, was sure to degenerate into indolent or sloughing sores, varying according to the peculiar idiosyncrasy and constitutional taint of the patient, several of which proved very obstinate and tedious of cure. I will take the liberty of detailing one case that recently came under my observation, for the purpose of showing how easily the surgeon may be perplexed, baffled, and perhaps disappointed in the effect and result of his most reliable remedies. One of our men, a landsman, aged 22 years, of a highly strumous habit, got a slight injury upon the leg, but did not report until some ten days after, when I found a large, perfectly round and deep indolent ulcer; it was of a black appearance, and situated in front, between the tibialis anticus and soleus muscles, and extending to the subcutaneous tissue of the tibia. I commenced with my usual plan of treatment. At the expiration of about ten days I had made use of all the effective remedies at hand, without even changing the condition or appearance of the ulcer in the least. My suspicious were aroused, and I ordered a strict watch over the patient, when I soon found that he was in the practice of removing the dressing and filling the ulcer with common salt, for the purpose, as he said, of preventing a healing process. I placed him in a straight jacket so he could not meddle with it, and in a few days' time, with ordinary treatment, the ulcer was quite well.

As I wish to be brief, I will conclude by remarking that "although my experience as a Naval Surgeon," is by no means extensive, yet I think there can be no doubt, and perhaps others have observed a predominant influence prevailing at sea, to bring forward, hasten, and mature strumous, tuberculous diathesis. My attention was called to the subject first from three cases of *Naval Officers*, laboring under incipient phthisis, and whom I was ordered to survey. Each of these cases had been exposed to this influence from a year to eighteen months, when as I assume, these *latent tubercles* upon the lungs were aroused into action, with spitting of blood and the usual premonitory symptoms of premature consumption. These cases were sent home probably to die, or perchance recover for a season by a timely change of air and climate. Such patients are often sent to sea with a view to their restoration; and it is a well conceded fact, that they either die or recover quite speedily. Now it is my belief, that if we could positively know the condition of the lungs of those who fortunately return home cured, or improved, we would find no tubercular development, nor did there any exist. On the other hand, if tubercles did exist, there is no improvement, but an aggravation, and speedy termination of the disease.

Most respectfully,

J. F. NORTON, M. D.,

Surgeon U. S. Brig Perry.

